# Nasal and ocular amyloidosis in a 15-year-old horse

**DOI:** 10.1186/s13028-014-0050-6

**Published:** 2014-08-27

**Authors:** Liv Østevik, Gjermund Gunnes, Gustavo A de Souza, Tale N Wien, Randi Sørby

**Affiliations:** Department of Basic Sciences & Aquatic Medicine, Faculty of Veterinary Medicine and Biosciences, Norwegian University of Life Sciences, N-0033 Oslo, Norway; Department of Immunology and Transfusion Medicine, Oslo University Hospital, University of Oslo, N-0027 Oslo, Norway; Department of Nephrology, Oslo University Hospital, N-0424 Oslo, Norway

**Keywords:** Localized amyloidosis, Nasal, Cornea, Horse, AL amyloid

## Abstract

Localized nasal, conjunctival and corneal amyloidosis was diagnosed in a 15-year-old pony with nasal and conjunctival masses and severe dyspnoea. Multiple swellings had been evident in the nostrils for at least two years and had gradually increased in size before presentation due to dyspnoea and exercise intolerance. Surgical debulking of the masses was performed and histological examination revealed large amounts of extracellular, hyaline, eosinophilic, Congo red positive material in the lamina propria of the nasal mucosa. A tentative diagnosis of localized nasal amyloidosis was made. The treatment relieved the clinical signs, however, the nasal masses recurred and bilateral conjunctival, papillary masses developed. The horse was euthanized. Nodular nasal and papillary conjunctival masses consisting of rubbery, grey to yellow tissue were found at necropsy. At the limbus this tissue infiltrated and expanded the cornea. The masses consisted of amyloid and moderate infiltrates of T lymphocytes and B lymphocytes were present in the tissue. No predominance of either cell type was observed and no distinct neoplastic mass could be identified. Ultrastructural examination of the nasal mucosa and cornea confirmed the presence of abundant extracellular deposits of non-branching fibrils ranging from 9–11 nm in diameter consistent with amyloid. Immunohistochemistry of amyloid revealed no labelling for AA amyloid, and no peptides representing serum amyloid A (SAA) were detected by microscopic laser dissection and subsequent mass spectrometry. Peptides from immunoglobulin kappa-like light chains were detected and are suggestive of AL amyloidosis, however the results were inconclusive and a final identification of the amyloid protein could not be made.

Nasal amyloidosis is a clinical entity of localized amyloid deposits in the horse. Localized amyloidosis involving the conjunctiva of the horse is previously described in only seven cases and the present case is the first case of combined, localized nasal and corneal amyloidosis in the horse. In several reported cases surgical excision has provided clinical improvement and return to normal levels of exercise, while medical treatment has had no effect. The present case however, shows that rapid recurrence and progression of nasal amyloidosis to involve ocular tissues can occur and lead to recurrent respiratory obstruction.

**Electronic supplementary material:**

The online version of this article (doi:10.1186/s13028-014-0050-6) contains supplementary material, which is available to authorized users.

## Background

Amyloidosis is a group of disorders characterized by extracellular deposits of hyaline, proteinaceous material which shows characteristic green birefringence under polarized light after staining with Congo red. The different amyloid proteins all form fibrils of beta pleated sheet conformation that are resistant to degradation and hence accumulate in tissues. Amyloid deposits are classified according to the type of protein deposited and as localized or systemic based on tissue distribution [1]. Several forms of amyloidoses are reported in animals, however in the horse only amyloid light chain (AL) amyloidosis and amyloid A (AA) amyloidosis have been described [1,2]. AA amyloidosis occur secondary to chronic inflammatory conditions where AA protein, a modified form of the acute phase protein, serum amyloid A (SAA), is deposited in major organs. In the horse the AA amyloid deposition preferentially occurs in the liver, but the spleen, kidneys, adrenals, gastrointestinal tract and lymph nodes may all be affected [2]. Both systemic and localized AL amyloidosis may occur in horses [3–19]. Localized amyloid deposition in horses primarily affects nasal tissue or skin, and nasal AL amyloidosis is a clinical entity in the horse [8–17]. Localized amyloidosis involving the conjunctiva of the horse is previously described in seven cases [12,16,18,19], two of which had concurrent localised nasal or multifocal, localised, upper and lower respiratory tract amyloidosis [12,16]. Equine corneal amyloid deposition is reported only once, in a horse with initial nodular cutaneous amyloidosis [18]. To the best of our knowledge the present case is the first equine case of combined, localized nasal, conjunctival and corneal amyloidosis. The purpose of this article is to describe the rare condition of localized ocular and nasal amyloidosis in the horse and provide a brief review of the literature.

## Case presentation

A 15-year-old Norwegian Shetland pony mare was presented to an equine clinic with marked dyspnoea and bilateral nodular and diffuse swelling of the nasal mucosa at the mucocutaneous junction and rostral nasal concha in June 2008. The nodular and diffuse swelling had been present for at least two years and had gradually increased in size and infiltrated the circumference of the nares to cause markedly reduced luminal diameter of both nostrils. Due to the respiratory difficulties surgical debulking of the tissue was performed. Histological examination of the excised tissue revealed large amounts of extracellular, hyaline, eosinophilic, Congo red positive material in the lamina propria of the nasal mucosa and a tentative diagnosis of localized nasal amyloidosis was made. The surgical treatment improved the clinical signs; however the nodular swellings recurred over the next months and multiple, papillary to nodular, conjunctival proliferations also developed bilaterally. In March 2009 the owners elected to euthanize the pony due to the recurrence of the nodular swellings and a reduced general condition of the animal as the swellings rapidly increased in size. The pony was euthanized and immediately necropsied.

**Figure 1:**
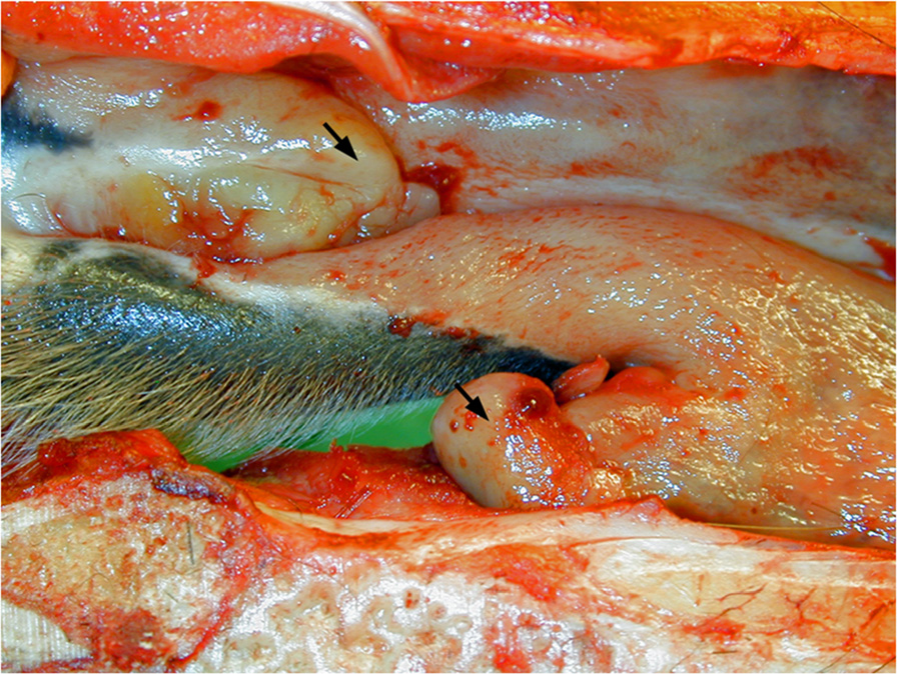
**Amyloid masses in the right rostral nasal cavity.** Nodular to diffuse thickening of the nasal mucosa at the mucocutaneous junction were observed (arrows). The proliferative tissue was rubbery, yellow to white and poorly demarcated from the surrounding nasal mucosa.

**Figure 2:**
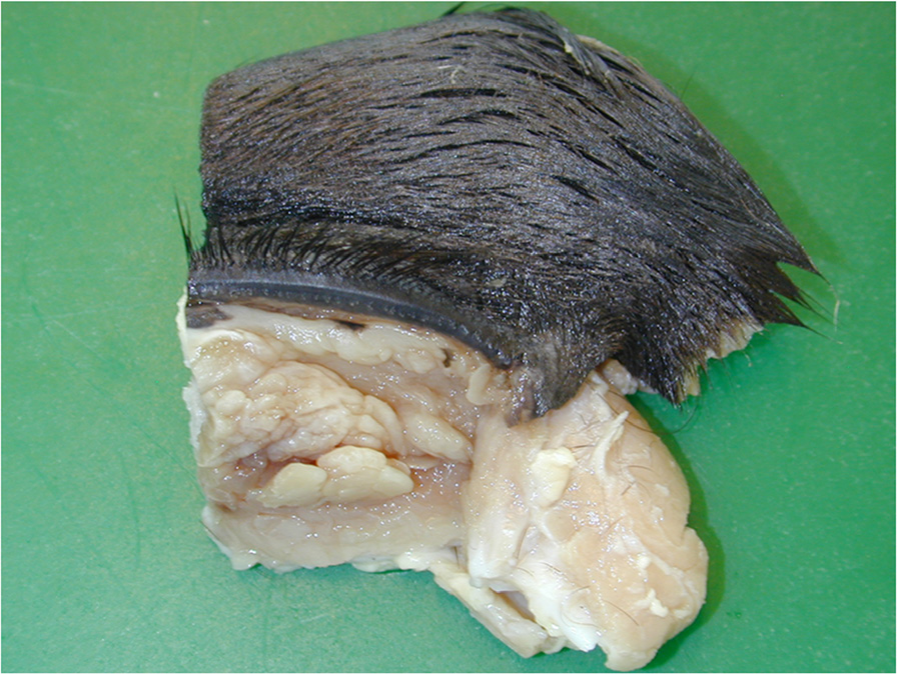
**Multiple papillary projections are present in the conjunctiva.** Irregular, papillary proliferations of the ocular conjunctiva were evident.

The mare was in normal body condition. A bilateral, nodular to diffuse thickening of the nasal mucosa at the mucocutaneous junction was evident (Figure 1). A nodule measuring 2×1×1 cm was present in the right nostril, as well as diffuse thickening of the rostral nasal mucosa. Similar milder changes were observed in the left nostril. There were bilateral, irregular, papillary proliferations in the ocular conjunctiva (Figure 2) and intense conjunctival hyperaemia. The proliferative tissue was rubbery, yellow to white and was protruding above the eyelid margins expanding the conjunctiva, causing marked disruption of the normal contour of the eyelids. At the limbus the cornea was expanded by a similar pale tissue. No other gross abnormalities were noted. Tissue samples for histology were collected from grossly abnormal tissue including the ocular globe, conjunctivae, and nasal mucosa, as well as sections from myocardium, lung, liver and kidney. Tissue samples were fixed in 4% buffered formaldehyde, routinely processed, embedded in paraffin, sectioned at 2 μm and stained with haematoxylin and eosin. Sections from the cornea, conjunctivae, nasal mucosa, liver and kidney were also stained with Congo red according to Pucthler *et al*. [20].

Immunohistochemistry was performed with the EnVision method using polyclonal rabbit anti-horse antibodies specific for AA amyloid (Anti amyloid K-10^a^) in 1/100 dilution with phosphate buffered saline and bovine serum albumin. Liver from a hyper-immunized horse used for serum production with confirmed hepatic and systemic amyloidosis served as positive control. Immunohistochemistry to characterize the inflammatory infiltrate was performed with polyclonal rabbit anti-human CD3 (A-0452, Dako, Glostrup, Denmark) and monoclonal mouse anti-human CD79 αcy (M0755, Dako) antibodies. Equine lymph node was used as positive control tissue. For negative controls addition of the primary antibody was omitted. The slides were counterstained with haematoxylin, dehydrated, and mounted in polyvinyl alcohol mounting medium.

For transmission electron microscopy selected areas of paraffin embedded samples from the nasal mucosa and cornea was deparaffinised with xylene, post-fixed with 2% osmium and re-embedded in LR-white. Counterstaining was performed with 4% aqueous uranyl acetate and 1% KMNO_4._

Samples for mass spectrometry (MS) were collected from formalin fixed paraffin embedded (FFPE) corneal and nasal tissue by laser microdissection as described in Additional file 1. Collection of only amyloid, identified as Congo red positive extracellular material, was attempted. Liver from the serum producing horse with systemic AA amyloidosis served as positive control for AA amyloid (data not shown). The collected material was subjected to protein identification using MS and a FFPE antigen retrieval approach [21]. MS raw files were submitted to MaxQuant software version 1.4.0.5 [22] for peptide and protein identification. Identification of peptides was based on parent ion mass and unequivocal fragmentation spectra.

**Figure 3:**
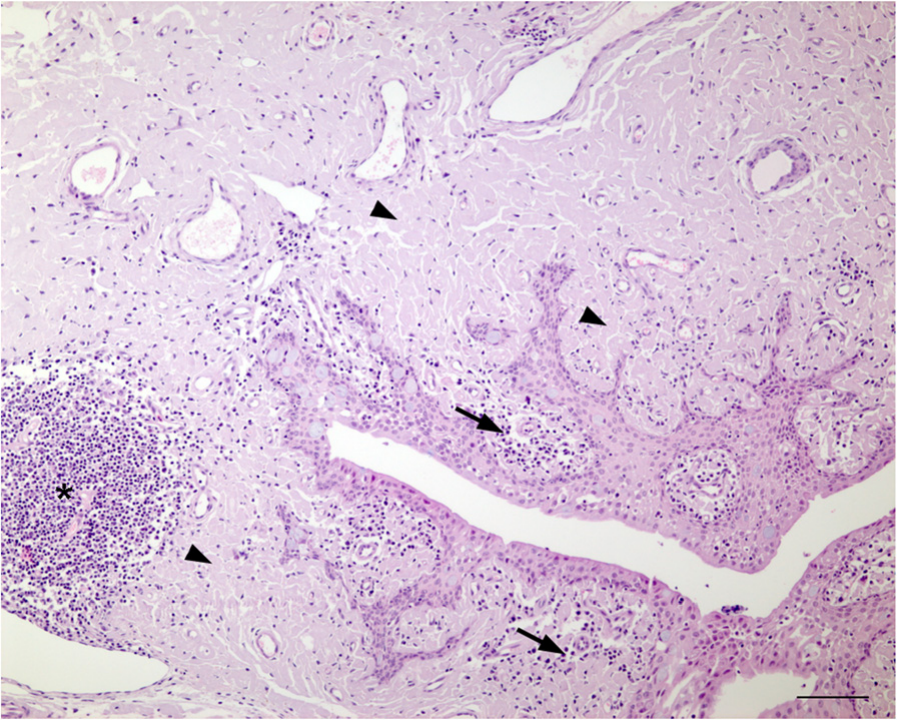
**Microscopic image of the conjunctiva.** Multifocal to coalescing aggregates of a hyaline, eosinophilic material were present in the lamina propria of the conjunctiva (arrowheads). The papillary structures consisted of lamina propria covered by hyperplastic, irregular epithelium. Multifocal, mild to marked, subepithelial and perivascular infiltrates of plasma cells and lymphocytes (arrows) occasionally forming large nodular aggregates (*) were present in the lamina propria. Haematoxylin and eosin stain; bar 100 μm.

**Figure 4:**
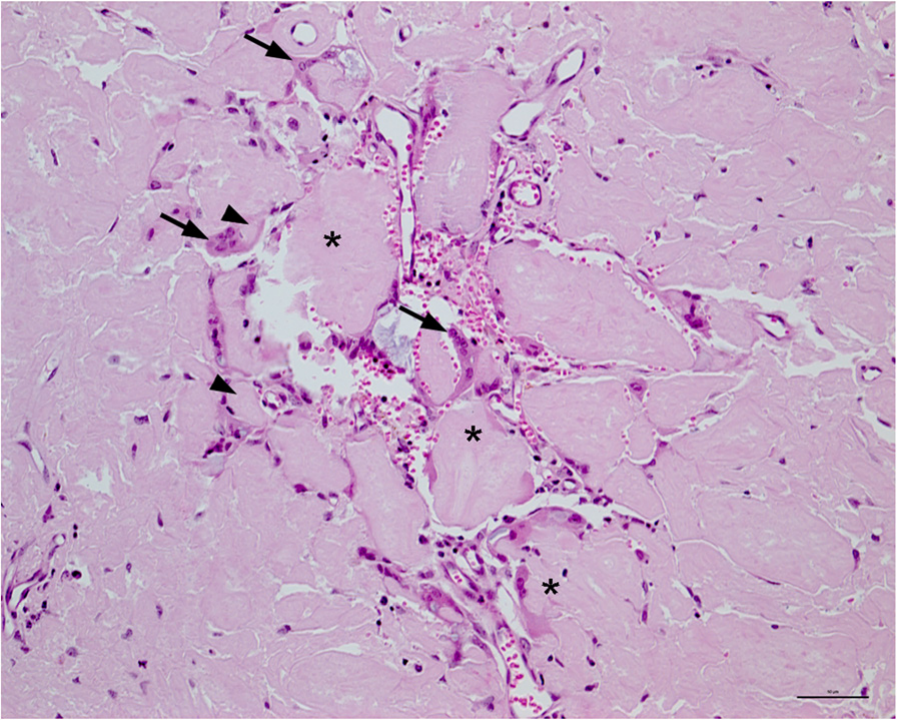
**Amyloid deposits and inflammatory infiltrates in the cornea.** Multifocal clusters of multinucleated giant cells (arrows), macrophages, lymphocytes and plasma cells surrounded the deposits in the cornea (*). Macrophages and giant cells appeared to have phagocytised amyloid (arrowheads). Corneal neovascularisation was also evident. Haematoxylin and eosin stain; bar 20 μm.

Microscopic examination confirmed the presence of multifocal to coalescing aggregates of a hyaline, eosinophilic material in the lamina propria of the nasal mucocutaneous junction, in the conjunctiva and in the corneal stroma (Figure 3). Hyaline, eosinophilic material was observed in the conjunctiva and nasal mucosa as large aggregates expanding the lamina propria, in a narrow subepithelial zone, in the interstitium of subepithelial glands of the nasal mucosa and around vessels. Occasionally hyaline deposits were present in the wall of arteries and arterioles. Large aggregates of hyaline, eosinophilic material were present in the superficial and middle aspect of the limbal corneal stroma. Multifocally, mild infiltrates of plasma cells, lymphocytes, macrophages, and occasional clusters of multinucleated giant cells were observed in the deposits in the cornea and conjunctiva. Macrophages and giant cells seemed to phagocytise the hyaline material (Figure 4). In the conjunctiva of the eyelids and the globe papillary structures consisting of lamina propria covered by hyperplastic, irregular epithelium was evident. Multifocal, moderate, subepithelial and perivascular infiltrates of plasma cells and lymphocytes occasionally forming nodular aggregates were present in the lamina propria of the nasal mucosa and conjunctiva. Mild subepithelial infiltration of plasma cells and lymphocytes was present in the limbal cornea. Additional findings in the conjunctiva included mild, multifocal infiltrates of partially degenerate neutrophilic granulocytes, intraepithelial pustules and neutrophil exocytosis.

**Figure 5:**
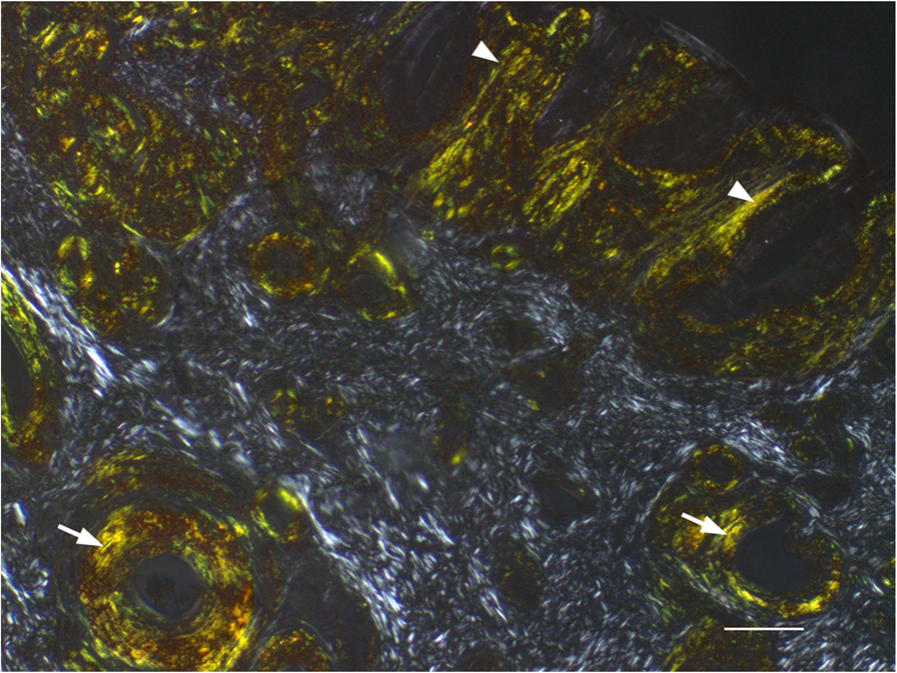
**Nasal amyloid display apple green birefringence in polarized light.** Multifocal to coalescing aggregates of hyaline, eosinophilic material in the lamina propria of the nasal mucocutaneous junction were observed perivascularly (arrow) and in a narrow subepithelial zone (arrowheads). The hyaline, eosinophilic material stained positive with Congo red and displayed apple green birefringence in polarized light. The collagen fibres of the lamina propria show white birefringence. Congo red stain; bar 100 μm.

The hyaline, eosinophilic material in both the nasal tissue, conjunctiva and cornea stained strongly with Congo red and displayed apple green birefringence in polarized light consistent with amyloid (Figure 5). No significant lesions or homogenous, eosinophilic, extracellular material suggestive of amyloid were detected in other examined organs and no Congo red positive material was observed when examining liver and kidney sections in polarized light.

The amyloid in the nasal mucosa and cornea was negative for anti-AA amyloid antibodies. The lymphocytes infiltrating the conjunctiva and nasal mucosa consisted of a mix of CD3^+^ T cells and CD79^+^ B cells. No clear predominance of either cell population was observed.

**Figure 6:**
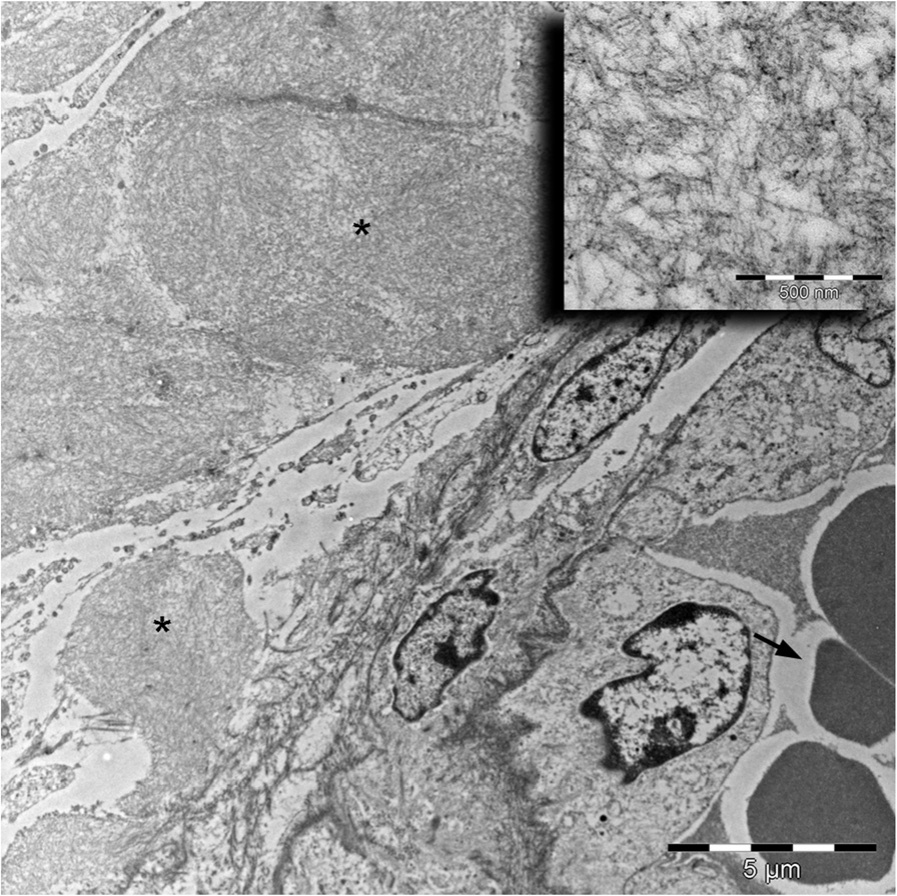
**Transmission electron microscopy the cornea revealed abundant amyloid deposits.** Surrounding a corneal blood vessel (arrow) was perivascular, extracellular non-branching fibrils (*). The fibrils ranged from 9–11 nm in diameter consistent with amyloid. Inset shows fibrils at high magnification. Bar 5 μm, inset; bar 500 nm.

Ultrastructural studies of both the nasal mucosa and cornea confirmed the presence of abundant extracellular deposits of straight non-branched fibrils ranging from 9–11 nm in diameter consistent with amyloid (Figure 6) [1].

Mass spectrometry data did not show a clear identification of the amyloid protein in any of the samples. Several immunoglobulin kappa-like peptides were detected among the most abundant proteins, but also albumin and hemoglobin were high on the list, indicating serum contamination of the sample (Additional file 2: Table S1 and Additional file 3: Table S2). Proteins from two other known amyloid precursor proteins, apolipoprotein A1 and apolipoprotein A4, were among the fifteen most abundant proteins identified in nasal and corneal samples. We also detected low amounts of peptides of other known amyloid precursor proteins, including lambda-like light chains, gelsolin, lysozyme and fibrinogen.

## Conclusions

The histological and immunohistochemical findings in this case are highly suggestive of localized, nasal and ocular, amyloid light chain amyloidosis. The lack of immunoreactivity to AA amyloid antibodies and the fact that no SAA peptides were detected by mass spectrometry suggests that a SAA protein origin is very unlikely. Lambda chains predominate in normal horse serum [23] with a reported kappa lambda ratio of approximately 0.08 [24,25]. The kappa abundance in samples analyzed by MS indicates overrepresentation of kappa light chains in the specimen, indicating a diagnosis of localized light amyloidosis of kappa type, alternatively a monoclonal gammopathy of kappa type with serum contamination of the specimens. Unfortunately, no serum samples were analyzed to exploit this further. At present only AA and AL amyloidosis have been described in the horse, however, with 30 different amyloid proteins identified in man [1], the possibility of a non-AA and non-AL amyloidosis in the horse should also be considered.

Localized, nasal amyloidosis is a rare clinical entity in the horse with only 23 reported cases (Table 1). Equine idiopathic, localized, nasal amyloidosis also involving the conjunctiva is rare, and amyloid deposition in the cornea in this entity has not been described previously. A horse described by Geisel *et al*. [18] developed unilateral corneal and conjunctival amyloidosis after initial presentation with nodular cutaneous amyloidosis. Over a period of 8 months after initial diagnosis this horse developed extensive, multifocal amyloid deposits involving not only skin, but also muscle, subcutaneous fat, nasal mucosa, the joint capsule and tendon of the tarsal joint, and was diagnosed with extramedullary plasmacytoma [18]. In both our case and the case described by Geisel *et al*. [18] amyloid deposition most likely affected the ocular structures by extension and progression of the amyloid disease.

**Table 1:** **Reported equine cases of localized nasal and ocular amyloidosis**

**Year**	**Authors**	**No cases**	**Type of amyloid**	**Method of amyloid typing**	**Locations**			**Location**	**Clinical signs**	**Concurrent diagnosis**	**Treatment (T)**
					**Eye**	**Nose**	**Other**				**Outcome (O)**
**1942**	**Hjarre and Nordlund** **[** 12 **]**	**3**	**Unknown**	**ND**	**1**	**3**	**3**	**1 case larynx, guttural pouches, pharynx, trachea, lungs (bronchii), submaxillary, retropharyngeal, mesenterial and thoracic lymph nodes, conjunctiva 1 case larynx and local lymph nodes 1 case larynx**	**Dyspnoea and epistaxis**	**Case 1: Acute purulent bronchopneumonia**	**Euthanasia**
**1927 to 70**	**Saunders and Rubin** **[** 19 **]**	**3**	**Unknown**	**NR**	**3**			**Conjunctiva**	**NR**	**NR**	**NR**
1987	Shaw *et al*. [8]	4	Unknown. Presumed AA	PS		4	1	1 case pharynx	Dyspnoea, reduced performance, respiratory stertor		T: Topical and systemic cortisone - no effect, then surgical excision,
											O: Returned to former use, no recurrence for 1 year
1988	Nappert *et al*. [11]	2	Unknown. Presumed AL	PS		2			Dyspnoea, epistaxis		T: Topical cortisone,
											O: No effect - euthanasia - one case. One case: NR
1988	Van Andel *et al*., Niewold *et al*.# [14,17]	6	AL	PS, IHC* Case 1WB AAS WB, AAS		6	1	Case 1- nodules subcutaneously, within fasciae and muscle, superficial lymph nodes, oesophagus, synovium, ileocaceal valve, thoracic cavity	Dyspnoea, stertor, epistaxis, wasting	Case 1: Histolymphocytic lymphosarcoma	T: Case 1; none,
											O: Euthanasia, Case 1,Case 2–6: NR
1989	Linke and Trautwein [13]	2	AL	IHC		2			Dyspnoea		T: Surgical excision
											O: NR
**1990**	**Mould** ***et al*** **.** **[** 16 **]**	**1**	**AL**	**WB, PS**	**1**	**1**			**Epistaxis and blood tinged epiphora**		**T: Surgical excision,**
											**O: Clinical relief, no recurrence 9 month follow-up**
1994	Kasper *et al*. [10]	2	Unknown, Presumed AL (1) AA (1)	PS		2			Dyspnoea, nasal discharge, respiratory stertor, episodic epistaxis		T: 1 case surgical excision, 1 case N,
											O: Improvement of clinical symptoms, no recurrence 12 month follow up
2012	Portela *et al*. [9]	1	Unknown	ND		1			Epistaxis		T: Surgical excision,
											O: Stable, but not in training
2012	Axiak and Johnson [15]	1	Unknown	ND		2			Exercise intolerance	NR	T: 1 case surgical excision, 1 case NR,
											O: Improvement of clinical symptoms, unknown follow-up
**2014**	**Østevik** ***et al*** **. (this publication)**	**1**	**Unknown, Presumed AL**	IHC*	**1**	**1**			**Dyspnoea, epiphora, conjunctivitis**	**Gastrophilus sp. infestation of gastric mucosa**	**T: Surgical excision,**
											**O: Recurrence amyloid and extension to cornea and conjunctiva - euthanasia**

Cases of localized ocular and combined nasal and ocular (conjunctival) amyloidosis are in bold. Cases of ERU, in which the hallmark lesion is ciliary body amyloid deposits, are not included. PS - Potassium permanganate sensitivity, IHC - immunohistochemistry for AL amyloid, IHC*- immunohistochemistry for amyloid A amyloid, WB - western blot, ND - not done, AAS - amino acid sequencing, NR - not reported. #Amyloid from case 1 in the paper by van Andel *et a*l. [14] is further characterized by Niewold *et al*. [17] and this case is therefore not included as separate case in this table.

In humans, only a single case of combined corneal and conjunctival amyloidosis has been reported in the literature. In this case the amyloid deposits were believed to be secondary to previous ocular trauma; however neither the corneal or conjunctival amyloid deposits were subjected to any form of typing [26]. Localized conjunctival AL amyloidosis is a rare condition in humans, and appears to be an entity distinct from localized nasopharyngeal and laryngo-tracheobronchial amyloidosis [27–29]. Ocular amyloidosis limited to the cornea secondary to chronic inflammation, such as trichiasis and keratoconus, are rare conditions in humans, and the precursor protein in these cases are lactoferrin produced by the corneal epithelium [30]. Additionally localized corneal amyloid deposition occurs in human gelatinous drop dystrophy and lattice dystrophies [1]. In these cases the amyloid precursor protein is lactoferrin in gelatinous drop dystrophy and kerato-epithelin or gelsolin in lattice dystrophies [1].

Immunohistochemistry and mass spectrometry are used to type amyloid in human medicine, and equine amyloid light chain deposits have been shown to cross-react with some anti-human light chain antibodies, however not consistently [5,13]. Typing of AL amyloid by immunohistochemistry is very challenging also in man. False negative results when using commercial light chain antibodies which frequently recognize epitopes on the constant portion of the light chain [31] is a major problem. There is a vast variability from case to case in light chain proteins which are highly modified. Additionally some authors report damage to AL deposits by formalin fixation [32], nonspecific antibody absorption of antibodies may occur due to amyloid “stickiness” [33] and the formalin fixation process can trap abundant normal serum protein in the tissue and amyloid deposits, leading to nonspecific background positivity, (“contamination”) [31,33]. Contamination can also be a problem in MS, causing identification of irrelevant “background" proteins as seen in our case. Lastly, in very rare cases, co-deposition of more than one amyloid protein has been reported [34].

Clinical signs in this case consisted of dyspnoea and epiphora and are most likely related to the space-occupying effect of the nodular amyloid deposits. In other cases of conjunctival and nasal amyloidosis epistaxis, blood tinged tears, respiratory stertor, nasal obstruction and exercise intolerance have been reported [8,11,12,14,16]. The masses of amyloid lead to nasal obstruction, with abnormal and reduced airflow causing both stertor and reduced exercise tolerance. Bleeding is believed to be a consequence of increased fragility of the mucosa in the affected areas leading to erosions and ulcerations, as well as loss of vascular integrity due to amyloid infiltrating blood vessels [35]. Conjunctivitis was observed in both our case and the case described by Geisel *et al*. [18] and may be secondary to physical disruption of the normal eyelid function by the extensive amyloid deposits. The amyloid fibrils are reported to have toxic properties, however ocular amyloidosis in humans is generally not associated with inflammation or pain and this is unlikely to be the cause of the conjunctivitis.

Primary localized conjunctival, as well as localized nasopharyngeal and laryngo-tracheobronchial AL amyloidosis has been described in humans [27–29,36]. Amyloid deposition may be non-progressive or progressive, and symptomatic surgical debulking is the treatment of choice and will often be successful in relieving the clinical signs [27,28,36]. The condition is considered benign and prognosis for survival is good to excellent, however the condition is often incurable and recurrence of amyloid deposits is noted in up to 50% of patients after surgical resection of the affected tissue [27,36]. Progression to systemic amyloidosis develops extremely rarely, but deaths due to severe haemorrhage of affected airways, as well as respiratory failure and recurrent airway obstruction have been reported in human patients with amyloidosis of the respiratory tract [27,37–39].

Surgical debulking of the affected nasal mucosa was performed and initially provided clinical relief in the pony mare. Recurrence occurred relatively rapidly and amyloid deposits also developed in the conjunctivae and cornea prompting the owners to euthanize the horse. This contrasts with previous results as surgical treatment in eight equine cases improved the clinical signs and horses successfully returned to previous levels of performance [8–10,15,16]. No recurrence or increase in the remaining amyloid deposits were observed, however follow up time was limited to 12 months in all cases. Additionally, these cases may not be representative because in many of the equine cases (10/23) outcome was not reported [10,11,13–15] and five horses were euthanized without treatment or after unsuccessful medical treatment [11,12,14].

Progression to systemic AL amyloidosis has not been reported in the horse, however the localized nasal amyloidosis in horses seems to be less strictly limited to the respiratory tract, compared to people, where nasal and conjunctival amyloidosis are distinct entities [27,28]. One horse initially presented with nasal amyloid deposits, subsequently developed T-cell rich B-cell lymphoma (histiolymphocytic lymphosarcoma) and amyloid deposition in multiple tissues, but parenchymal organs remained unaffected [14]. Additionally, nasal amyloidosis in horses can progress to involve the ocular structures [12] and progression of conjunctival amyloid to involve the nasal mucosa has been reported in a single horse [16]. Three equine cases with sole affection of conjunctival tissues are also described, however no information regarding development of the condition and outcome are reported [19].

## Endnote

^a^Donated by G. Husby Professor Emeritus of Medicine, Institute of Clinical Medicine, University of Oslo, Oslo, Norway.

## Additional files

Additional file 1:
**Mass spectrometry method.**
Additional file 2: Table S1. Top 15 most abundant proteins from the cornea as identified by mass spectrometry. Immunoglobulin kappa-like proteins, apoplipoprotein A1 (APOA1) and apolipoprotein A4 (APOA4) are among the most abundant proteins identified in the sample. However, high amounts of hemoglobin indicative of serum contamination was also detected. Additional file 3: Table S2. Top 15 most abundant proteins from nasal mucosa as identified by mass spectrometry. Immunoglobulin kappa-like proteins and apoplipoprotein A1 (APOA1) are among the most abundant proteins identified in the sample. However, high amounts of hemoglobin and albumin indicative of serum contamination was also detected.

## Abbreviations

AA: Amyloid A
AL: Amyloid light chain
FFPE: Formalin fixed paraffin embedded
MS: Mass spectrometry
SAA: Serum amyloid A
