# Supplementary material for: Nasal and ocular amyloidosis in a 15-year-old horse
Source: Acta Vet Scand. 2014 Aug 27;56(1):50. doi: 10.1186/s13028-014-0050-6 (PMC4223893; doi:10.1186/s13028-014-0050-6)
Supplement: Additional file 1: — Mass spectrometry method. [file 13028_2014_50_MOESM1_ESM.doc]

**Additional file 1: Mass spectrometry method**

Formalin fixed paraffin embedded tissue from the Shetland pony mare and the control case with hepatic amyloidosis were examined by mass spectrometry. Briefly, paraffin embedded tissue sections were sectioned at 10 µm and placed on separate membrane slides for laser micro-dissection (No. 50103, Molecular Machines and Industries, Zürich – Glattburg, Switzerland). The tissue samples were routinely stained with Congo red but not mounted with cover slips. The membranes were rinsed with absolute alcohol and allowed to air-dry in room temperature for 10 minutes. Laser microdissection was performed with SLµCut micro-dissection system (Molecular Machines and Industries). Amyloid was identified as Congo red positive material in the light microscope. Nasal, corneal and liver amyloid was collected in separate tubes (Molecular Machines and Industries).

The collected material was submitted for protein identification using mass spectrometry and a FFPE amyloid retrieval approach (Vra*na et a*l., 2009). Briefly, tissue pieces are washed with 30 µL TE buffer pH 7.4 and heated at 98^o^C for 90 min. Sample is spin down every 10 min to avoid evaporation in the lid. Sample is then sonicated for 60 min for proteins extraction. Proteins are reduced using 10mM DTT in 50mM ammonium bicarbonate, incubated at 56^o^C for 30 min, followed by alkylation with 55 mM iodacetamide in 50mM ammonium bicarbonate, RT for 20 min in the dark. Peptides are obtained by enzymatic digestion using 0.05µg of trypsin (modified grade, Promega) and overnight incubation at 37^o^C. Resulting peptides were desalted on microcolumns using C18 empore extraction disks (Varian, St. Paul, MN, USA). The peptides were analyzed on a Dionex Ultimate 3000 nano-LC system (Dionex, Sunnyvale, CA, USA) which was connected to a quadrupole-Orbitrap (QExactive) mass spectrometer (ThermoElectron, Bremen, Germany) equipped with a nanoelectrospray ion source (Easy Spray, Thermo). An Acclaim PepMap100 RSLC column (C18, 2µm beads, 100Å, 75µm inner diameter) (Dionex, Sunnyvale, CA, USA) of 25 cm bed length was used to separate the peptides. The flow rate used was 0.3µl/min and the solvent gradient was 5 to 32% B in 120 min (solvent A: 0.1% formic acid, solvent B: 90% ACN/0.1% formic acid) and then 32 to 45% B in 20 min. The mass spectrometer was operated in the data-dependent acquisition mode using the Xcalibur 2.2 software. Single MS full-scan in the Orbitrap (300–1750 m/z, 70000 resolution at m/z 200, AGC target 3e6, maximum IT 50 ms) were followed by 10 data-dependent MS/MS scans in the Orbitrap after accumulation of 1e5 ions in the C-trap or an injection time of 100 ms at 17500 resolution (isolation width 3.0 m/z, underfill ratio 5 %, dynamic exclusion 30 s). The normalized collision energy was set to 25%. The sample was run in triplicate.

MS raw files were submitted to MaxQuant software version 1.4.0.5 (Cox and Mann, 2008) for peptide and protein identification. Pyro-glu (N-term Q and N-term E) and oxidation (M) were set as variable modifications, carbamydomethilation (C) was used as fixed modification. Mass error allowed during first search was 20ppm and after mass recalibration the main search error was set to 6ppm. Trypsin without proline restriction was selected and two miscleavages were allowed. Minimal unique peptides were set to 1, and a false discovery rate of 0.01 (1%) was used in all instances. Identification of peptides was based on parent ion mass and unequivocal fragmentation spectra.

References

Cox J, Mann M (2008) MaxQuant enables high peptide identification rates, individualized p.p.b.-range mass accuracies and proteome-wide protein quantification. *Nature Biotechnology,* **26**, 1367-1372.

Vrana JA, Gamez JD, Madden BJ, Theis JD, Bergen HR, III *et al.* (2009) Classification of amyloidosis by laser microdissection and mass spectrometry-based proteomic analysis in clinical biopsy specimens. *Blood,* **114**, 4957-4959.
