# Supplementary material for: Nasal and ocular amyloidosis in a 15-year-old horse
Source: Acta Vet Scand. 2014 Aug 27;56(1):50. doi: 10.1186/s13028-014-0050-6 (PMC4223893; doi:10.1186/s13028-014-0050-6)
Supplement: Additional file 2: Table S1. — Top 15 most abundant proteins from the cornea as identified by mass spectrometry. Immunoglobulin kappa-like proteins, apoplipoprotein A1 (APOA1) and apolipoprotein A4 (APOA4) are among the most abundant proteins identified in the sample. However, high amounts of hemoglobin indicative of serum contamination was also detected. [file 13028_2014_50_MOESM2_ESM.docx]

| **Peptide Counts (unique)** | **Gene symbol** | **Protein Ids** | **Unique Sequence Coverage [%]** | **Mol. Weight [kDa]** | **iBAQ** |
| --- | --- | --- | --- | --- | --- |
| 19 | APOA1 | F6Z2L5 Uncharacterized protein | 66.9 | 30.33 | 481510000 |
| 2 | Ig kappa-like | F6SQD7 Uncharacterized protein (Fragment) | 7.5 | 11.226 | 97551000 |
| 32 | ALB | F6SQD7 Uncharacterized protein (Fragment) | 59.5 | 68.372 | 96337000 |
| 3 | LOC100052889 | F6PWV1 Histone H2B, | 26.2 | 13.922 | 51151000 |
| 2 | Ig kappa-like | F6SP11 Uncharacterized protein (Fragment) | 27.4 | 10.98 | 45746700 |
| 3 | Ig alpha-like | H9GZU9 Uncharacterized protein (Fragment) | 16.3 | 35.84 | 24404400 |
| 6 | HIST2H4A | F6VFV9 Histone H4 | 46.6 | 11.367 | 19090100 |
| 9 | HBB | P02062b Hemoglobin subunit beta | 65.3 | 16.139 | 18019300 |
| 23 | APOA4 | F6RZ27 Uncharacterized protein | 53.5 | 43.251 | 15174500 |
| 3 | HTRA1 | F7B812 Uncharacterized protein | 11.1 | 35.149 | 13600700 |
| 4 | Ig alpha-like | H9GZT5 Uncharacterized protein (Fragment) | 10.4 | 36.432 | 13127000 |
| 10 | CLU | Q29482 Clusterin | 21.2 | 52.153 | 11112500 |
| 3 | LOC100063021 | F6X6J0 Histone H2A (Fragment) | 25.9 | 14.522 | 9966000 |
| 5 | IGHC1 | H9GZQ9 Uncharacterized protein (Fragment) | 25.8 | 37.437 | 9325600 |
| 3 | HBA | P01958 Hemoglobin subunit alpha | 35.2 | 15.245 | 9161500 |

**Additional file 2: Table S1 - Top 15 most abundant proteins from the cornea as identified by mass spectrometry**

Immunoglobulin kappa-like proteins, apoplipoprotein A1 (APOA1) and apolipoprotein A4 (APOA4) are among the most abundant proteins identified in the sample. However, high amounts of hemoglobin indicative of serum contamination was also detected
