# Supplementary material for: Nasal and ocular amyloidosis in a 15-year-old horse
Source: Acta Vet Scand. 2014 Aug 27;56(1):50. doi: 10.1186/s13028-014-0050-6 (PMC4223893; doi:10.1186/s13028-014-0050-6)
Supplement: Additional file 3: Table S2. — Top 15 most abundant proteins from nasal mucosa as identified by mass spectrometry. Immunoglobulin kappa-like proteins and apoplipoprotein A1 (APOA1) are among the most abundant proteins identified in the sample. However, high amounts of hemoglobin and albumin indicative of serum contamination was also detected. [file 13028_2014_50_MOESM3_ESM.docx]

| **Peptide Counts (unique)** | **Gene symbol** | **Protein Ids** | **Unique Sequence Coverage [%]** | **Mol. Weight [kDa]** | **iBAQ** |
| --- | --- | --- | --- | --- | --- |
| 2 | Ig kappa-like | F6SQD7 Uncharacterized protein (Fragment) | 7.5 | 11.226 | 1100210000 |
| 19 | APOA1 | F6Z2L5 Uncharacterized protein | 66.9 | 30.33 | 1039430000 |
| 2 | Ig kappa-like | F6SP11 Uncharacterized protein (Fragment) | 27.4 | 10.98 | 459460000 |
| 9 | HBB | P02062 Hemoglobin subunit beta | 65.3 | 16.139 | 450370000 |
| 32 | ALB | P35747 Serum albumin | 59.5 | 68.372 | 412349000 |
| 3 | HBA | P01958 Hemoglobin subunit alpha | 35.2 | 15.245 | 344321000 |
| 23 | APOA4 | F6RZ27 Uncharacterized protein | 53.5 | 43.251 | 307711000 |
| 6 | HIST2H4A | F6VFV9 Histone H4 | 46.6 | 11.367 | 249100000 |
| 3 | LOC100063021 | F6X6J0 Histone H2A (Fragment) | 25.9 | 14.522 | 196501000 |
| 5 | LOC100059034 | F6V881 Uncharacterized protein | 10.7 | 54.236 | 161459000 |
| 3 | LOC100052889 | F6PWV1 Histone H2B | 26.2 | 13.922 | 146906000 |
| 16 | VIM | F7B5C4 Uncharacterized protein (Fragment) | 38.5 | 42.143 | 99976000 |
| 3 | Ig alpha-like | H9GZU9 Uncharacterized protein (Fragment) | 16.3 | 35.84 | 97661000 |
| 9 | BGN | O46403 Biglycan | 31.7 | 41.924 | 91535000 |
| 65 | COL6A3 | F6R735 Uncharacterized protein | 25 | 342.14 | 77552000 |

**Additional file 3: Table S2 - Top 15 most abundant proteins from nasal mucosa as identified by mass spectrometry**

Immunoglobulin kappa-like proteins and apoplipoprotein A1 (APOA1) are among the most abundant proteins identified in the sample. However, high amounts of hemoglobin and albumin indicative of serum contamination was also detected.
